# Peripheral neural cell sensitivity to mTHPC-mediated photodynamic therapy in a 3D *in vitro* model

**DOI:** 10.1038/sj.bjc.6605197

**Published:** 2009-07-28

**Authors:** K E Wright, E Liniker, M Loizidou, C Moore, A J MacRobert, J B Phillips

**Affiliations:** 1 Life Sciences Department, The Open University, Milton Keynes MK7 6AA, UK; 2 University College London Medical School, University College London, 67-73 Riding House Street, London W1W 7EJ, UK

**Keywords:** PDT, Foscan, neuron, satellite glial cell, MCF-7 cell, 3D culture

## Abstract

**Background:**

The effect of photodynamic therapy (PDT) on neural cells is important when tumours are within or adjacent to the nervous system. The purpose of this study was to investigate PDT using the photosensitiser, meta-tetrahydroxyphenyl chlorin (mTHPC), on rat neurons and satellite glia, compared with human adenocarcinoma cells (MCF-7).

**Methods:**

Fluorescence microscopy confirmed that mTHPC was incorporated into all three cell types. Sensitivity of cells exposed to mTHPC-PDT (0–10 *μ*g ml^–1^) was determined in a novel 3-dimensional collagen gel culture system. Cell death was quantified using propidium iodide and cell types were distinguished using immunocytochemistry. In some cases, neuron survival was confirmed by measuring subsequent neurite growth in monolayer culture.

**Results:**

MCF-7s and satellite glia were significantly more sensitive to PDT than neurons. Importantly, 4 *μ*g ml^–1^ mTHPC-PDT caused no significant neuron death compared with untreated controls but was sufficient to elicit substantial cell death in the other cell types. Initially, treatment reduced neurite length; neurons then extended neurites equivalent to those of untreated controls. The protocol was validated using hypericin (0–3 *μ*g ml^–1^), which caused neuron death equivalent to other cell types.

**Conclusion:**

Neurons in culture can survive mTHPC-PDT under conditions sufficient to kill tumour cells and other nervous system cells.

Photodynamic therapy (PDT) uses photosensitive drugs (photosensitisers), combined with light and in the presence of molecular oxygen to cause tissue destruction. A range of photosensitisers have been developed, some of which have been used clinically for cancer treatment. One such photosensitiser (meta-tetrahydroxyphenyl chlorin (mTHPC) Foscan, Biolitec AG, Jena, Germany) has been reported to be effective in destroying tumour cells, although not causing major damage to the peripheral nerves in experimental models (Kubler *et al*, 2003) and in clinical practice (Ris *et al*, 1996; Lou *et al*, 2004; Betz *et al*, 2007). It is not yet clear whether this relative nerve sparing is a feature particular to mTHPC or shared with other photosensitisers. Indeed, PDT using topical administration of another photosensitiser, 5-aminolevulinic acid, is associated with pain, perhaps through a direct interaction with peripheral nerve endings in skin undergoing treatment (Stender *et al*, 2006; Cottrell *et al*, 2008). A photosensitiser that enables PDT to be used effectively to destroy tumours but spares nerve tissue would be a valuable tool in the treatment of tumours whose location makes nerve damage a likely side effect of traditional surgical treatments. However, for this therapeutic benefit to be fully exploited, the cell biology underlying the effects of mTHPC-mediated PDT needs to be delineated.

There have been relatively few studies exploring the effects of PDT on isolated cells of the peripheral nervous system. Most studies of PDT on peripheral nervous system cells have used non-mammalian models such as crayfish neurons, which provide a useful system in which to compare photosensitisers, and monitor their effects on neuronal function. Uzdensky *et al* showed that in preparations of stretch receptors from crayfish, neuronal conduction was inhibited after PDT mediated by mTHPC at concentrations as low as 10 nM (with 633 nm light at fluence rate 0.3 W cm^–2^) (Uzdensky *et al*, 2004). In separate studies with a different photosensitiser the same group also attempted to explore the effects of interactions between neurons and glia on PDT sensitivity (Lobanov and Uzdensky, 2005).

Neuron–glia interaction is of fundamental importance when investigating the effects of PDT on nerves. Peripheral nerves comprise the axons of sensory and motor neurons, which are accompanied by about ten times as many glial cells providing metabolic and structural support (Hall, 2005). The glial cells along the length of the peripheral nerves are termed Schwann cells and those surrounding the neuronal cell bodies in the ganglia are satellite cells (Jessen, 2004). Neurons and glia are intimately related throughout the nervous system, and the level of complexity surrounding their responses to each other means that, when considering *in vitro* models, it is often more appropriate to use cocultures of neurons and glia rather than try to study either cell type in isolation.

In this study the aim was to investigate the effect of mTHPC-mediated PDT on mammalian peripheral nerve cells. A novel cell culture model was developed in which primary neurons and satellite glia from dissociated rat dorsal root ganglia (DRGs) were cocultured in collagen gels. Collagen gel systems have been used previously to model peripheral nerves (Phillips *et al*, 2005) and have advantages over conventional monolayer cultures for some investigations (Brown and Phillips, 2007). Here the reason for using collagen gels was that they trap cells, which die during PDT treatment, enabling accurate quantification of cell death; this is in contrast to monolayer cultures, in which the dead cells are more easily washed away, and can therefore be lost for quantification purposes. Furthermore, relatively thin collagen gels were used to facilitate illumination of cells during PDT treatment and permit subsequent analysis of cell survival in conjunction with immunofluorescence to distinguish neurons from glia. Monolayer systems were also used where appropriate for visualisation of photosensitiser uptake and quantification of neurite length.

These model systems enabled accurate assessments to be made of the sensitivity of neurons and glia to mTHPC-mediated PDT using a well-established cancer cell line (MCF-7) as a comparator.

## Materials and methods

### Cell cultures

Neurons and satellite glial cells were cultured from DRGs, twenty of which were isolated from each freshly culled 250–350 g Sprague–Dawley rat as previously described (Phillips *et al*, 2005). Using microscopic dissection, DRGs were removed, cleaned of all nerve processes and incubated for 90 min at 37°C in Dulbecco's Modified Eagle's Medium (DMEM) with L-glutamine containing 0.125% collagenase (Sigma, St Louis, MO, USA), then triturated to produce a cell suspension. Culture medium (DMEM supplemented with 10% fetal calf serum (FCS) and 1% penicillin/streptomycin) was added and cells were separated from the collagenase by centrifugation for 5 min at 100 **g** and resuspended in an appropriate volume of culture medium. Dorsal root ganglion cells included both neurons and satellite glia. The tumour cells used in this study were the human breast adenocarcinoma cell line, MCF-7 (ECACC, Dorset, UK), used at passage 305–321.

MCF-7 cells were cultured in 75-cm^2^ flasks in fully supplemented DMEM until 95% confluent whereupon they underwent routine trypsinisation (0.25% trypsin–EDTA, 5 min, 37°C). Cells were washed by centrifugation for 5 min at 100 **g** and resuspended in an appropriate volume of culture medium.

### Cell culture models

Two-dimensional (2D) and three-dimensional (3D) cell culture models were used in this study. Photosensitiser uptake and neurite length experiments were conducted using cells in 2D cultures, whereas cell death assays used 3D cultures in order to trap non-adherent cells that would have been lost from 2D cultures following treatment.

For 2D cell culture, neural cells and MCF-7 cells were grown on glass coverslips coated with 20 *μ*g ml^–1^ poly-L-lysine (PLL, Sigma). Cells were seeded in 100 *μ*l of suspension (1–2 × 10^5^ cells per ml), allowed to adhere for 30 min, then incubated in 1 ml culture medium in 12-well culture plates.

For 3D cultures used for cell death assays, 5–7 × 10^5^ DRG or MCF-7 cells were suspended in 0.6 ml culture medium, then added to a solution containing 4.8 ml type I rat tail collagen (2 mg ml^–1^) that had been mixed with 0.6 ml 10 × Minimal Essential Medium and neutralised with 1 M NaOH. This mixture was then quickly dispensed into 24-well plates, 200 *μ*l per well, and formed a gel within 5 min at 37°C. Gels were covered with 1 ml culture medium and maintained in culture at 37°C, 5% CO_2_, for 4 days (DRG cells) or 1 day (MCF-7 cells) before experimentation. Pilot studies showed no difference in response to mTHPC-PDT between MCF-7 cells cultured for 1 or 4 days (data not shown); the 1-day option was chosen here to minimise time for proliferation of these rapidly dividing cells.

### Photodynamic therapy

Culture medium was removed from coverslips or gels and cultures were incubated with photosensitiser drug. In all cases, mTHPC (Biolitec AG) or hypericin (Tocris Cookson Ltd, Bristol, UK) was added to the cultures in medium that contained no phenol red indicator or FCS (colourless DMEM supplemented with 1% penicillin/streptomycin and 1% L-glutamine), and plates were incubated for 4 h (mTHPC) or 7 h (hypericin) in the dark at 37°C, 5% CO_2_. The medium containing photosensitiser was then removed, and the cultures were washed with phosphate-buffered saline (PBS) before fresh colourless culture medium (supplemented with 10% FCS) was added, and the cultures were then exposed to light. The drug incubation in colourless media and PBS washes also served to remove any traces of phenol red indicator from the collagen gels before light exposure.

For experiments investigating the effect of mTHPC on neurite length in 2D neuronal cultures, blue light from a transilluminator (LumiSource, PCI Biotech, Oslo, Norway) with a peak wavelength of 420 nm and a fluence rate of 7 mW cm^–2^ was used for 10 min, giving a total light dose of 4.2 J cm^–2^. For cell death experiments on cells in 3D cultures, white light from a transilluminator (Northern Light Illuminator, InterFocus Imaging Ltd, Cambridge, UK) with a fluence rate of 1.6 mW cm^–2^ was used for 10 min, giving a total light dose of 1 J cm^–2^. The minimal thickness (<1 mm), relatively low cell density and transparent nature of the collagen gels ensured that all cells within them received an equivalent light dose. Controls were included in each experiment that involved excluding either the drug (by using drug-free colourless medium during the incubation step) or the light (by wrapping the plate in foil during the illumination step) or both.

### mTHPC detection within cells

To assess whether photosensitiser had been incorporated into cells, the intrinsic fluorescence of mTHPC was detected using fluorescence microscopy. Cells were prepared as described and plated onto coverslips in 2D culture, incubated with 4 *μ*g ml^–1^ mTHPC for 4 h, washed in PBS and fixed in 4% paraformaldehyde at 4°C for 1 h. In some cases, cells were stained using immunocytochemistry as described below. Meta-tetrahydroxyphenyl chlorin fluorescence was detected using an Olympus BX61 fluorescence microscope (excitation at 595 nm, emission through long pass filter cut-on at 615 nm) and images were captured using a ColorView Soft Imaging System CCD camera and analySIS software (Olympus, Philadelphia, PA, USA). Confocal microscopy was performed in some cases using a Leica TCS-NT laser scanning microscope (Leica Microsystems, Wetzlar, Germany). In specific cases cells were labelled immunocytochemically as described below.

### Immunostaining

Immunostaining was carried out to distinguish neurons from satellite glial cells in the DRG cultures after PDT treatment. Neurons were detected using an antibody to *β*-III-tubulin, and satellite glia were detected using an antibody to S100 (Phillips *et al*, 2005). All dilutions and washes were carried out using PBS. Coverslips and gels were washed, then fixed in 4% paraformaldehyde for 1 h at 4°C, washed again, then permeabilised using 0.5% Triton-X-100 for 10 min, then washed and blocked with 5% swine serum (DAKO, Glostrup, Denmark) for 10 min. After washing, cells were incubated for 1.5 h in primary antibody, either mouse anti-*β*-III-tubulin (1 : 400, Sigma) or rabbit anti-S100 (1 : 200, DAKO) or both, washed, then incubated for 45 min in the appropriate anti-species secondary antibody (1 : 100, Sigma) conjugated to fluorescein isothiocyanate or tetramethylrhodamino isothiocyanate (TRITC) before final washing and mounting. Nuclei were stained using 10 *μ*M Hoechst 33258 (Sigma), which was included in the secondary antibody incubation.

### Effect of PDT on neurite length

For investigating the effect of PDT on neurite length in 2D culture, cells obtained from the DRGs of one rat were resuspended in 2.5 ml culture medium, then 100 *μ*l of this cell suspension was seeded onto 19 mm diameter glass coverslips and incubated in culture medium at 37°C in a 5% CO_2_ humidified atmosphere for 3 days before drug treatment.

To assess the ability of neurons to produce neurites after they had been subjected to PDT in 3D collagen gels, neurons were extracted from collagen at 24 h after PDT treatment by incubation with 0.125% collagenase for 30 min, which released the cells into suspension. Cells were then collected by centrifugation at 100 **g**, washed in culture medium, then mixed with a suspension of satellite glial cells, seeded onto PLL-coated 19-mm diameter coverslips and maintained in culture for 2 days before fixation and immunostaining. The satellite glial cells used for this stage were previously obtained from DRGs as described, but were expanded in PLL-coated flasks until confluent, then subjected to cycles of trypsinisation, transferred to new flasks, then further expanded to dilute the neuronal population (the neurons are postmitotic). During each cycle, confluent cultures were subjected to vigorous shaking to dislodge neurons, which were then discarded. The resulting satellite glial cultures contained no detectable neurons, as determined by immunostaining for *β*-III-tubulin in control samples (data not shown).

### Neurite length measurement

β-III-tubulin immunoreactivity was detected in DRG cultures on coverslips using a fluorescence microscope (Nikon Diaphot, Nikon, Tokyo, Japan) or Olympus BX61 (Olympus), and images were captured using a CCD camera (Hamamatsu Orca, Hamamatsu Photonics, Hamamatsu, Japan) or ColorView Soft Imaging System and Openlab (Improvision, Coventry, UK) or analySIS software. Total neurite length was measured on each coverslip by manually tracing all of the detectable neuronal processes and summing their lengths. For the investigation of neurite growth after PDT in 3D gels, one coverslip containing neurons from half a gel mixed with 1.2 × 10^4^ untreated satellite cells was used, all the neurons on each coverslip were assessed for the presence or absence of neurites, and the total neurite length was measured as before.

### Cell death analysis

Propidium iodide was used to determine the extent of cell death at 24 h after PDT in 3D cell cultures. Collagen gels were incubated with 200 *μ*g ml^–1^ propidium iodide in PBS for 10 min, then washed in PBS and fixed using 4% paraformaldehyde overnight. Gels were then immunostained to distinguish between neurons and glia as described above and analysed using fluorescence microscopy (Olympus BX61). In this way, dead cells could be distinguished from those that were alive before fixation by the presence of propidium iodide in their nuclei. The numbers of live and dead satellite glia and MCF-7 cells were determined by manually counting and scoring all cells in three 3D fields of each gel (typically a total of ∼160 cells per gel). Fields were chosen at random using a × 40 objective, and the operator performing the counting was blinded as to which treatment each of the gels had received. The operator focussed through all planes of the gel during the counting process so that each field was a cylindrical sample through the gel from top to bottom. Pilot experiments confirmed that sampling three fields in this way was sufficient to provide an accurate representation of the whole gel. There were considerably fewer neurons than satellite glia in each gel, hence all of the *β*-III-tubulin immunoreactive cells in each gel (typically up to 50 neurons) were identified and classified as being live or dead. For statistical analyses, live/dead cell counts from the three pooled 3D fields (MCF-7s and satellite glia) or all the neurons in each of the nine independent gels (mTHPC) or six independent gels (hypericin) were analysed using one-way analysis of variance (ANOVA) with Tukey's *post-hoc* test to compare groups. Data were displayed as mean % cell death to normalise differences in absolute cell number between experiments. Basal levels of cell death with and without light exposure are listed in the figure legends.

## Results

### Detection of mTHPC

#### mTHPC was detected in the cytoplasm of all cells

**Figure 1:**
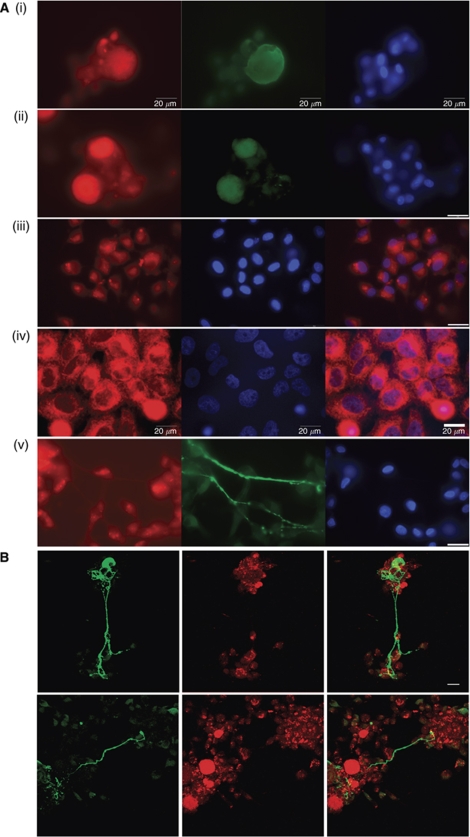
Representative micrographs showing (**A**) mTHPC fluorescence within the cell body of neurons (i and ii), satellite glia (iii) and MCF-7 cells (iv), but not detected in neurites (v). (**B**) Confocal micrograph projections confirming the presence of mTHPC fluorescence in neuronal cell body and satellite glia, but none detectable along the neurite. Meta-tetrahydroxyphenyl chlorin fluorescence is shown in red, *β*-III-tubulin immunoreactivity is shown in green, nuclei are blue. All scale bars are 20 *μ*m. Confocal images are maximum intensity projections from 40 optical sections in a 14 *μ*m z-series.

The presence of mTHPC in neurons, satellite glia and MCF-7 cells was detected using fluorescence microscopy of 2D cultures (Figure 1A). mTHPC fluorescence was detected throughout the cytoplasm in each cell type but was not present in the nuclei or in the neurites (the axonal projections of neurons). Confocal microscopy was performed on DRG cultures to confirm the results of the fluorescence microscopy. Figure 1B shows neurites bridging areas of satellite glia, and although there is drug fluorescence in the cell bodies of the neuron and glia, none is detectable along the neurites.

### Effect of PDT on neurite length

#### mTHPC-mediated PDT reduced the length of neurites in 2D culture

**Figure 2:**
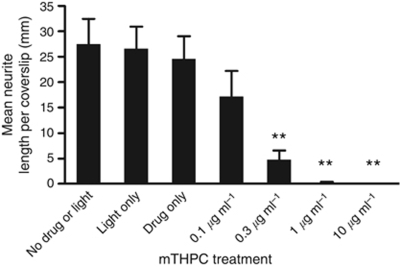
Reduction in neurite length due to mTHPC-mediated PDT. Dorsal root ganglion neurons were cultured on coverslips for 3 days, then treated with various concentrations of mTHPC and light, then maintained in culture for a further 24 h before fixation and quantification of the total length of all *β*-III-tubulin immunoreactive neurites. Data are means±s.e.m., *N*=4. (^**^P<0.001 for treatments compared with no drug or light control, one-way ANOVA with Dunnett's multiple comparison *post test*).

The effect of mTHPC-mediated PDT on neurite length in DRG cultures was tested in the 2D system. After 3 days in culture, cells were treated with mTHPC and light, and the effect of this treatment on neurite length was analysed 24 h later (Figure 2 and Supplementary Figure 2). There was no statistically significant effect on the neurite length when drug or light were applied separately, or when PDT was performed using 0.1 *μ*g ml^–1^ mTHPC, whereas PDT using higher concentrations of mTHPC (0.3 *μ*g ml^–1^ and above) caused a significant reduction in neurite length compared with untreated controls (*P*<0.001, one-way ANOVA with Dunnett's post test).

### Relative sensitivity of different cell types to mTHPC-PDT

#### Neurons showed lower sensitivity to mTHPC-mediated PDT than MCF-7s and glia

**Figure 3:**
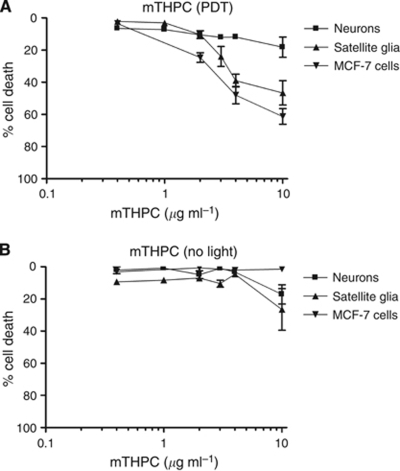
Relative sensitivity of cells to mTHPC-mediated PDT. Cell death (neurons, satellite glia and MCF-7 cells) in the presence of various concentrations of mTHPC after light exposure (**A**) or in control samples with no light exposure (**B**). Differences in cell death in neurons compared with MCF-7 cells and satellite glia were present at concentrations above 2 *μ*g ml^–1^ mTHPC, with ∼50% cell death for MCF-7 cells and satellite glia at 4 *μ*g ml^–1^. Neuronal cell death after this treatment was significantly lower at ∼12%, which was not different from untreated neurons (*P*<0.001 using one-way ANOVA with Tukey's post test). Data are means±s.e.m. of at least nine replicates. Basal levels of % cell death using 0 *μ*g ml^–1^ mTHPC with light were 3.7±0.8, 4.3±1.3 and 7.6±1.0, and with no light were 3.1±1.0, 4.2±1.4 and 1.89±0.7, for neurons, satellite glia and MCF-7s, respectively.

The extent to which mTHPC-mediated PDT caused cell death was explored in neurons, satellite glia and MCF-7s using a 3D culture system. The dose-response profile obtained showed a difference in sensitivity between the cell types, with neurons showing considerably less sensitivity to mTHPC-mediated PDT than the other cell types (Figure 3). In particular, at concentrations of 4 *μ*g ml^–1^, 48±5.4% of MCF-7 cells and 39±4.0% of satellite glia died compared with only 11.9±1.4% of neurons (*P*<0.001 using one-way ANOVA with Tukey's post test), which showed no more death than their untreated counterparts.

It is worth noting that the neuronal death that was detected was not significantly different from the amount detected in drug-only (no light) controls (Figure 3B) at concentrations up to and including 10 *μ*g ml^–1^ mTHPC (one-way ANOVA with Tukey's post test), nor was it significantly different from that detected after light treatment in 0 *μ*g ml^–1^ mTHPC controls.

#### No change in neural cell death at different times after PDT

**Figure 4:**
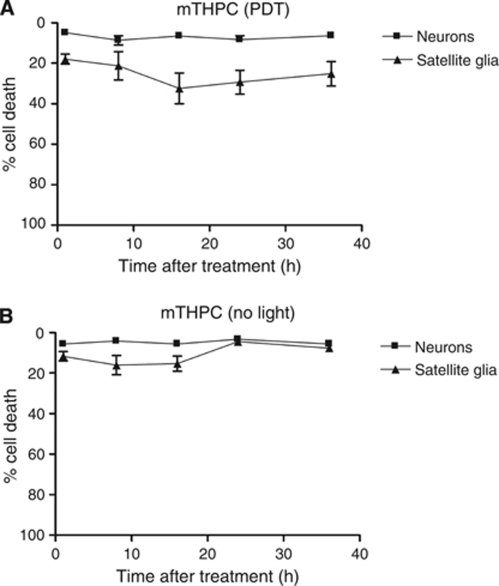
Time course of neuronal and satellite glial death after mTHPC-mediated PDT. Cells in 3D gels were incubated with 4 *μ*g ml^–1^ mTHPC for 4 h, exposed to light (**A**) or used as no-light controls (**B**), then maintained in culture for various times before cell death analysis and immunodetection of neurons.

In Figure 3, the extent of cell death at 24 h after PDT was used to show the differences in sensitivity between neurons and other cells. The time course for cell death after PDT can vary according to cell type, drug/light dose and various other parameters, so it was important to establish that the 24 h time point was appropriate for this study. Therefore, parallel cultures of neurons and satellite glia were treated using 4 *μ*g ml^–1^ mTHPC and maintained in culture up to 36 h. Figure 4 shows the time course of cell death after mTHPC-mediated PDT. There was no significant difference in the extent of cell death in the cultures analysed after different lengths of time.

### Relative sensitivity of different cell types to hypericin-PDT

#### Neurons, MCF-7s and glia were all sensitive to hypericin-mediated PDT

**Figure 5:**
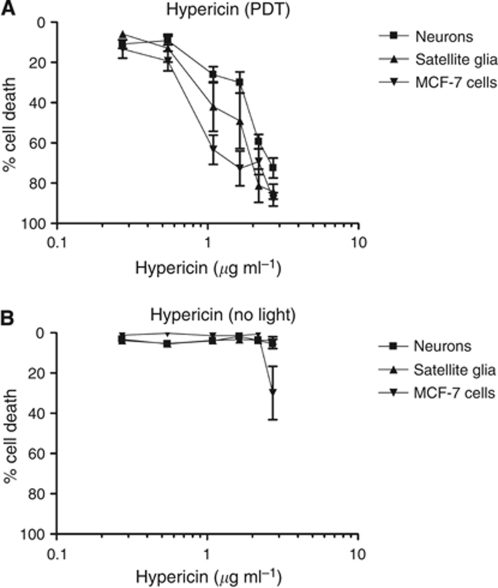
Relative sensitivity of cells to hypericin-mediated PDT. Cell death (satellite glia, neurons and MCF-7s) in the presence of various concentrations of hypericin after light exposure (**A**) or in control samples with no light exposure (**B**). Neurons showed a similar pattern of response as satellite glia and MCF-7 cells, with 50% cell death at ∼1–2 *μ*g ml^–1^. Data are means±s.e.m. of at least six replicates. The basal levels of % cell death using 0 *μ*g ml^–1^ hypericin with light were 7.9±1.1, 4.8±0.9 and 1.0±0.2, and with no light were 5.4±1.3, 8.0±2.8 and 1.6±0.7, for neurons, satellite glia and MCF-7s, respectively.

To investigate the sensitivity of the cells to another PDT agent and confirm that neuronal death could be detected using this model, the experiment was repeated using hypericin. As for mTHPC, hypericin fluorescence was detected throughout the cytoplasm in each cell type, but was not present in the nuclei or in the neurites (Supplementary Figure 1). Figure 5 shows that hypericin-mediated PDT resulted in neuronal death, following a similar dose-response pattern to that of satellite glia and MCF-7 cells, and at concentrations of 2.2 *μ*g ml^–1^ and above, there was a significant difference in cell death between hypericin-PDT-treated neurons and untreated controls (*P*<0.001 using one-way ANOVA with Tukey's post test).

### Functionality of neurons after mTHPC-PDT

#### Spared neurons were functional after mTHPC-mediated PDT

**Figure 6:**
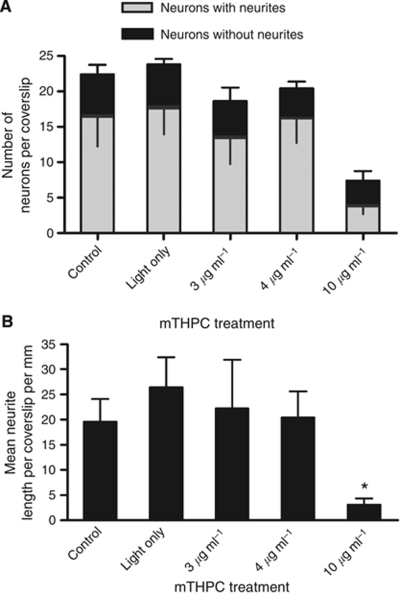
Neurons survive mTHPC-mediated PDT and produce new neurite outgrowth. After treatment with mTHPC-mediated PDT in 3D culture, neurons were grown in 2D in the presence of untreated satellite glia for 2 days. A similar number of neuronal cell bodies had neurites associated with them in the 3–4 *μ*g ml^–1^ mTHPC-mediated PDT samples as in the controls (**A**). There was a similar level of neurite length detected in controls and treated neurons, except for the samples that had received 10 *μ*g ml^–1^ mTHPC-mediated PDT, which showed a significant reduction in neurite length (^*^P<0.05) (**B**).

Having established that PDT with 3, 4 and 10 *μ*g ml^–1^ mTHPC resulted in a low level of neuronal death in the 3D culture system, the ability of ‘spared’ neurons to sprout neurites was investigated. After PDT, neurons (along with remaining glia and dead cells) were harvested from collagen gels using enzymatic digestion, mixed with untreated satellite glia, then seeded in a monolayer culture. Figure 6A shows the numbers of *β*-III-tubulin immunoreactive neuronal cell bodies that showed neurite growth at 2 days after transfer to monolayer culture. Similar numbers of neurons were detected in control, light only and 3 and 4 *μ*g ml^–1^ mTHPC-mediated PDT samples, although there were approximately one-third as many neurons detected in the 10 *μ*g ml^–1^ mTHPC-mediated PDT sample. There were similar numbers of neurons without neurite growth in all groups. Overall, it is apparent that neurons treated with 3 and 4 *μ*g ml^–1^ mTHPC-mediated PDT showed no difference to untreated and light-only controls in their ability to produce neurites.

Furthermore, when the length of neurite growth was quantified (Figure 6B and Supplementary Figure 3), there was no significant difference in the extent of neurite outgrowth between the control and light only, and 3 and 4 *μ*g ml^–1^ mTHPC-mediated PDT-treated cells, with only the 10 *μ*g ml^–1^ mTHPC-mediated PDT-treated cells showing significantly reduced neurite outgrowth compared with light-only control (one-way ANOVA with Dunnett's post test).

## Discussion

The key finding from this study is that DRG neurons were less sensitive to mTHPC-mediated PDT than their associated satellite glial cells and the tumour cell line, MCF-7, both of which exhibited dose-dependent cell death over the range of treatment concentrations used here. Importantly, photosensitiser concentrations of 3–4 *μ*g ml^–1^ mTHPC caused no dark toxicity but, when activated by light, yielded substantial glial and MCF-7 cell death as previously reported for MCF-7 cells in other culture systems (Koechli *et al*, 1995). At these concentrations, there was no detectable neuronal death above that seen in untreated controls. This is an important observation as it indicates that DRG neurons are able to survive PDT treatments that kill tumour cells, offering the possibility that therapeutic regimens could be developed to treat tumours in close proximity to neurons.

However, it is clear from this study that PDT using lower doses of mTHPC (0.3 *μ*g ml^–1^) can have a profound effect on DRG neurons growing in 2D cell culture, causing a substantial reduction in neurite length. This effect is reversible, however, since when neurons treated with 3 and 4 *μ*g ml^–1^ mTHPC-mediated PDT were cocultured with untreated glial cells, they exhibited the same level of neurite extension as untreated neurons. The ability of these treated neurons to extend neurites acts as an important indication that they remain functionally active following survival of PDT treatment in terms of their regenerative capacity.

It should be noted that previous studies showed that PDT using mTHPC was sufficient to inhibit conduction in crayfish stretch receptor neurons at concentrations much lower than those used in this study (10 nM, equivalent to 0.0068 *μ*g ml^–1^) (Uzdensky *et al*, 2004). These previous data, together with our neurite retraction results, indicate that, in cell culture, neuronal function is compromised by mTHPC-mediated PDT, but that unlike other cell types they have the capacity to survive PDT treatment with the potential for regeneration and subsequent restoration of function. It is interesting that the decrease in neurite length in 2D cultures occurs despite the lack of detection of any photosensitiser along the length of the neurites. It is possible that mTHPC was present in this narrow cellular region that could not be detected because of limitations in the resolution of the microscopy. This would be consistent with the lack of detection of the control photosensitiser hypericin in neurites under the same conditions. Alternatively, it could be that activation of mTHPC localised to the neural cell body is sufficient to cause a reduction in neurite length. Detailed investigation of the cellular localisation of mTHPC within neurons would be a useful future direction as differences in drug distribution between neurons and other cells might account for the differences in sensitivity reported here.

The accurate detection of the effects of mTHPC-mediated PDT on mammalian cell death in this study was made possible by the development of a bespoke culture system. This used a thin collagen gel as a support substrate for the cells under investigation, enabling a powerful combination of cell death analysis and cell identification to be carried out. Collagen gels have been used as culture substrates for many years because they provide a 3D environment in which cells can grow in a more natural spatial and physical environment than they would on a stiff monolayer substrate (Kim *et al*, 2004; Brown and Phillips, 2007). The rate of diffusion of oxygen and other nutrients through collagen gels is relatively rapid, facilitating cell survival under normal conditions and ensuring that oxygen availability does not become a limiting factor during PDT treatment. However, the main advantage of using a collagen gel system in this study was that cells remained trapped within the gels throughout treatment and in subsequent analysis stages. This meant that cell death could be determined accurately – in many studies in which conventional cell monolayers are used, a proportion of cells (particularly dead ones) are washed away during these stages, so the resulting analyses of cell death or cell viability (for example, live/dead staining, 3-(4,5-dimethylthiazol-2-yl)-2,5-diphenyl tetrasodium bromide (MTT) assay) do not account for all of the cells. Furthermore, trapping the cells in this manner enabled the multiple washes and incubations required for a propidium iodide assay to be used in conjunction with an immunostaining technique so that numbers of live or dead neurons and glial cells could be analysed separately within a coculture.

As the interaction between neurons and their associated glial cells is important for neuronal function and survival, coculture systems such as this are often favoured over isolated neuronal cultures. This is the first study in which the differential effects of mTHPC-mediated PDT have been shown on mammalian DRG neurons and glia in coculture. A previous study using isolated crayfish stretch receptors (neurons and glia) and the photosensitiser Photosens (a mixture of sulphonated alumophthalocyanines) mentioned that glial cells seemed more sensitive than neurons to proteolytic and PDT treatments in that system (Lobanov and Uzdensky, 2005). It would be interesting to explore whether mammalian neurons and glia have different sensitivities to Photosens in our system. The complex relationship between neurons and glia makes it difficult to speculate about a mechanism by which PDT reduced neurite length without killing neurons, as neurite outgrowth can be influenced by factors such as the level of activation of the supporting glia (Armstrong *et al*, 2008), membrane proteins expressed on the surface of the glia (Castro and Kuffler, 2006) and diffusible factors released from them (Armstrong *et al*, 2007). This means that the changes in neurite length measured in these cocultures could be a result of the effect of the PDT on the accompanying glia rather than a direct effect on the neurons. Therefore, it was important to culture the treated neurons with untreated glial cells in the experiments to determine the functionality of neurons after PDT, thus removing the influence the PDT-treated glia might exert on neurite growth. Remarkably, the neurons, which had been treated with PDT using 3–4 *μ*g ml^–1^ mTHPC, not only produced neurites, they did it in a manner indistinguishable from untreated and light-only treated controls, both in terms of the proportion of neuronal cell bodies detected that showed neurite outgrowth and in terms of their length. Neurite outgrowth is a well-established measure of neuronal behaviour in cell culture models using DRGs; so, this acts as a compelling indication that the neuronal survival detected in the cell death assays was genuine and that neurons survived the mTHPC-mediated PDT doses administered in this system.

In this study, hypericin was used as an alternative to mTHPC primarily to validate the culture system. The sensitivity of MCF-7 cells to hypericin-mediated PDT in this system was similar to that previously reported (Vandenbogaerde *et al*, 1997). The high level of neuronal death (∼90%) detected after hypericin-mediated PDT showed that the lack of neuronal cell death after mTHPC-mediated PDT was not due to an inability to detect neuronal death in this system. Together, the mTHPC and hypericin data show that this system can be used to compare mixtures of cells in coculture, exposing them to PDT and then combining cell death analysis with immunofluorescence identification to dissect out any differences in sensitivity between the cell populations. This is a valuable model which could facilitate research in which the response of two or more cell types to PDT needs to be evaluated and may be particularly useful for studies comparing responses in tumour cells and non-tumour cells during screening for selective photosensitisers.

The results presented here indicate that there are particular mTHPC-mediated PDT conditions in which neurons survive treatments that kill tumour cells and glia. This observation needs to be followed up by animal studies to explore whether this phenomenon occurs *in vivo* before its potential for application in the clinical setting can be determined. Importantly, in the animal studies and human cases in which peripheral nerve sparing has been reported, the PDT was applied to the nerve trunk rather than to the ganglia containing the neuronal cell bodies (Ris *et al*, 1996; Kubler *et al*, 2003; Lou *et al*, 2004; Betz *et al*, 2007). Our *in vitro* study indicates that neurons might survive PDT directly applied to the ganglia if an appropriate mTHPC concentration and light dose were used, enabling PDT to be applied to areas of the body rich in neuronal cell bodies such as the DRGs without killing the neurons. An important consideration for the further investigation of neuronal sparing during PDT is the role of the vasculature. Nerve tissue is highly vascularised and its function is dependent on effective perfusion (Myers *et al*, 1986; Hogan, 2008). This feature of nerve tissue is not accounted for in cell culture and *in vivo* if neurons survived a PDT insult they might still be compromised by severe damage to their blood supply. It has been shown in rabbits that some blood vessels are relatively resistant to mTHPC-mediated PDT (Kubler *et al*, 2003), however, in studies investigating the effect of other photosensitisers, it has been difficult to determine whether nerve function is affected by direct damage to neurons or indirect damage to the vasculature supplying the neural tissue (Dole *et al*, 2005; Huang *et al*, 2007).

The potential for clinical application of a photosensitiser that showed relative nerve sparing would be particularly helpful in the prostate, where nerve damage during treatment for prostate cancer is related to loss of erectile function, and in cancers of the head and neck, which can lie in close proximity to nerves of great functional importance, and in the treatment of bone metastases in the spine, which can lead to effects on adjacent nerves. The model described here could allow the further pre-clinical investigation of different photosensitisers, which are being considered for clinical use in these areas.
