# Supplementary figures and images for: Peripheral neural cell sensitivity to mTHPC-mediated photodynamic therapy in a 3D in vitro model
Source: Br J Cancer. 2009 Jul 28;101(4):658–65. doi: 10.1038/sj.bjc.6605197 (PMC2736832; doi:10.1038/sj.bjc.6605197)

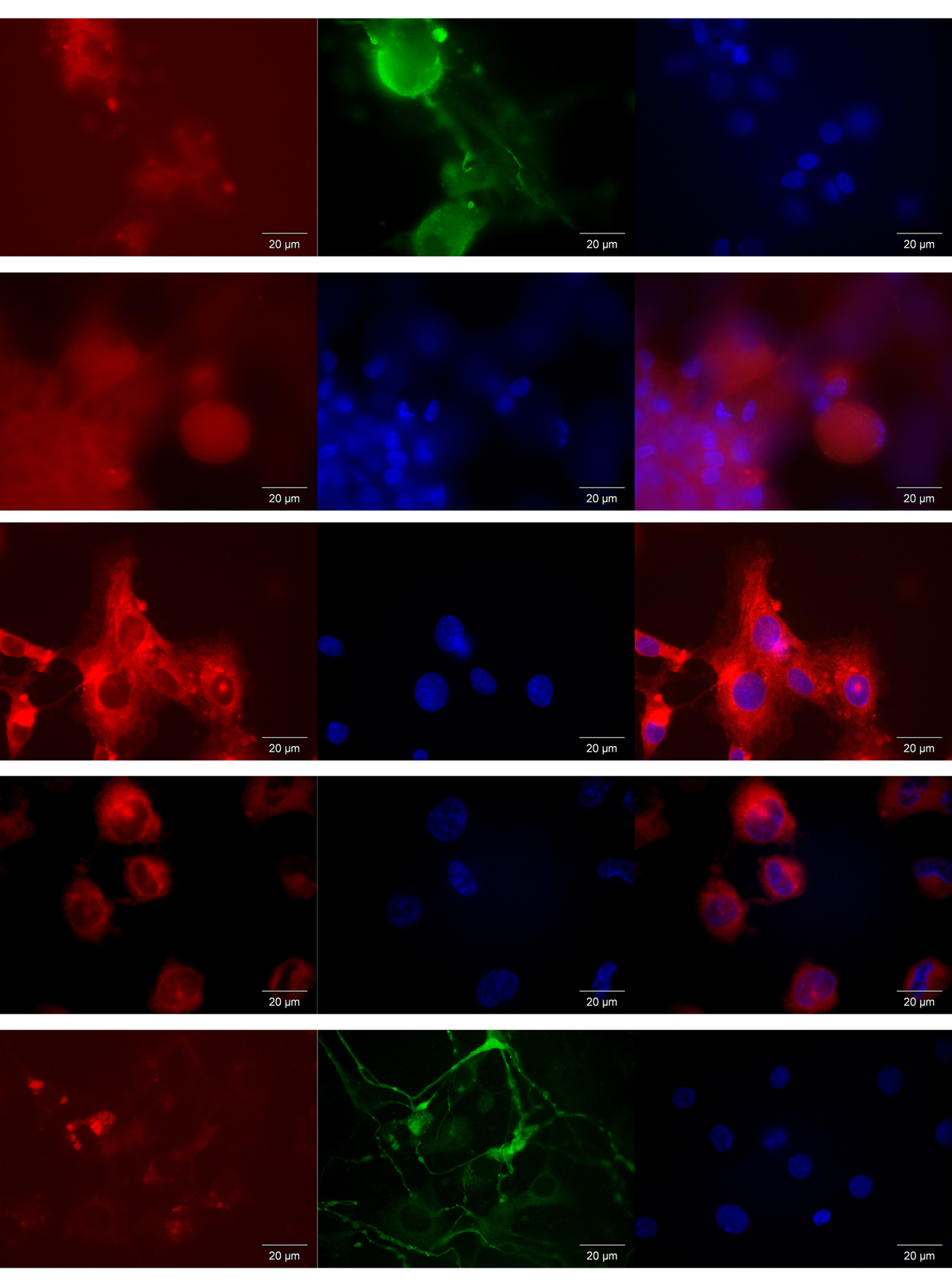

Supplement: Supplementary Figure 1 [file 6605197x1.jpg]

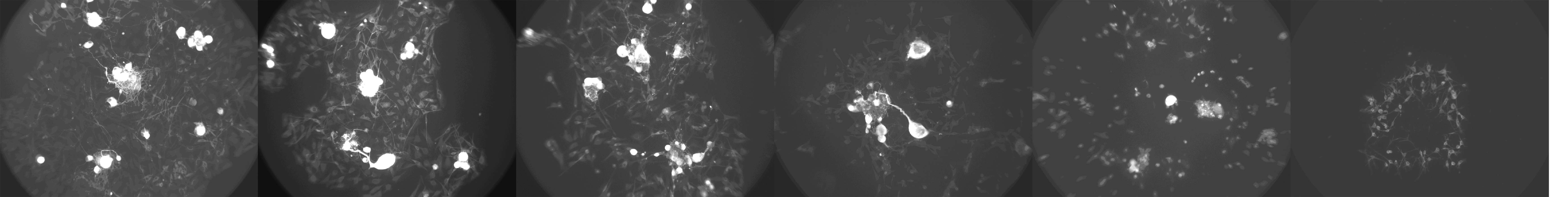

Supplement: Supplementary Figure 2 [file 6605197x2.gif]

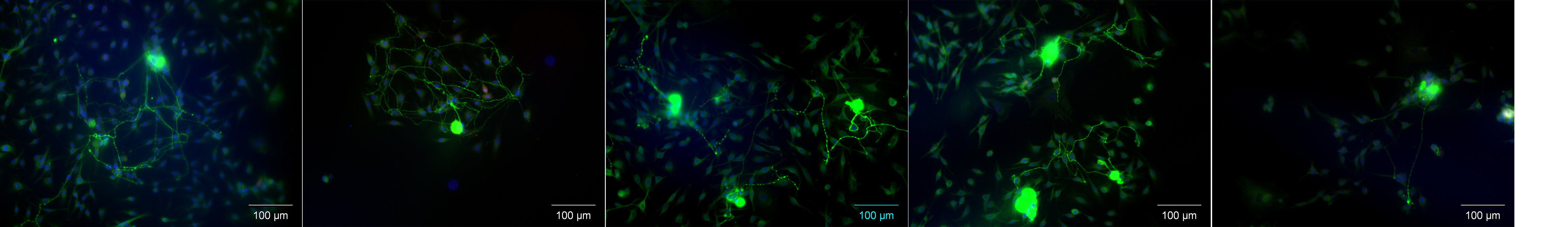

Supplement: Supplementary Figure 3 [file 6605197x3.jpg]

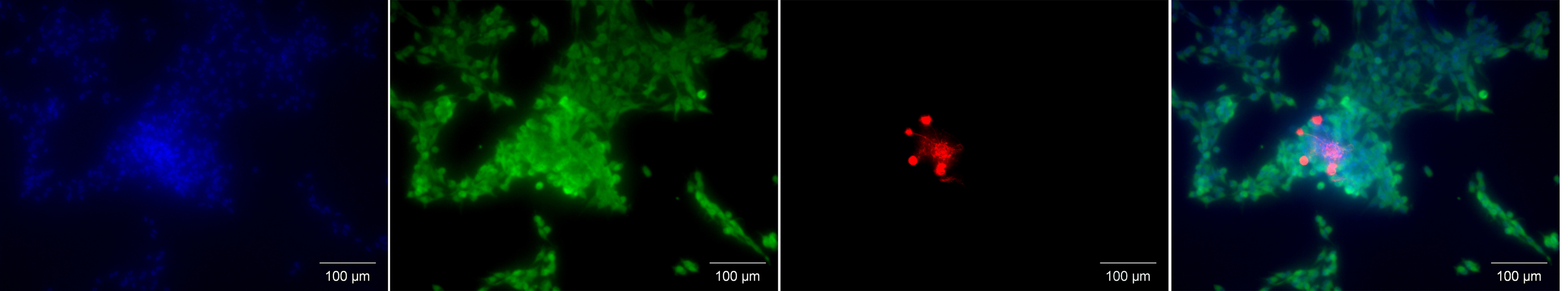

Supplement: Supplementary Figure 4a [file 6605197x4.jpg]

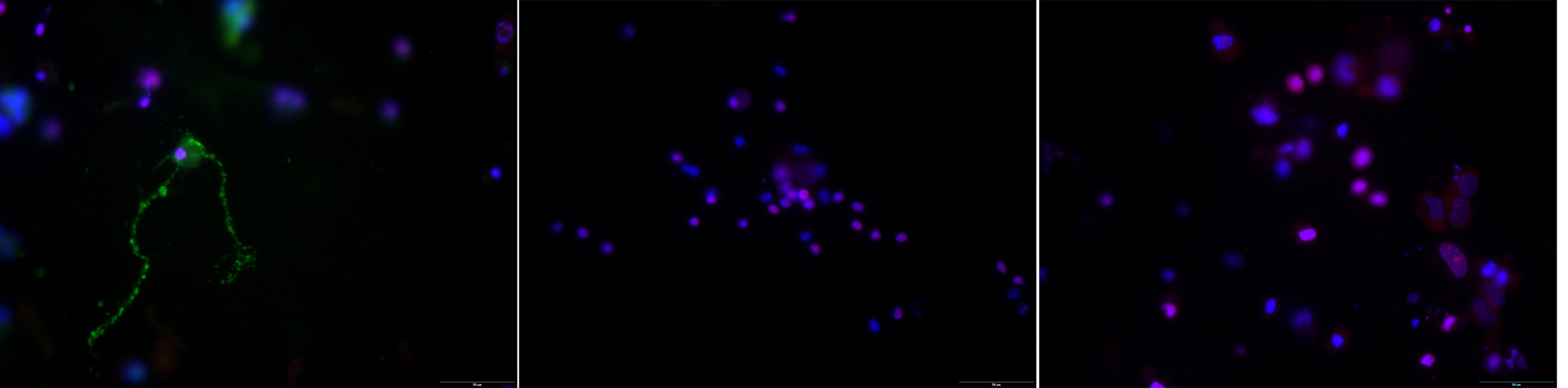

Supplement: Supplementary Figure 4b [file 6605197x5.jpg]
