# Supplementary material for: Peripheral neural cell sensitivity to mTHPC-mediated photodynamic therapy in a 3D in vitro model
Source: Br J Cancer. 2009 Jul 28;101(4):658–65. doi: 10.1038/sj.bjc.6605197 (PMC2736832; doi:10.1038/sj.bjc.6605197)
Supplement: Supplementary Figures Legends [file 6605197x6.doc]

**Supplementary Figure 1.** Representative micrographs showing hypericin fluorescence within the cell body of neurons (i) and, because the immunostaining process causes a reduction in hypericin levels, within the cell body of neurons that have not undergone immunostaining (ii). Hypericin fluorescence is also shown in satellite glia (iii) and MCF-7 cells (iv) but was not detected in neurites (v).

**Supplementary Figure 2.** Representative micrographs showing the effect of mTHPC-mediated PDT on the neurites of neurons growing in 2D culture. DRG neurons were cultured for 3 days then subjected to PDT with various doses of mTHPC, then maintained in culture for 24 h prior to immunodetection of βIII-tubulin. Images capture was optimised to enable measurement of neurite length. Micrographs displayed are single fields from cultures with either no treatment (i), or PDT treatment with 0 (ii), 0.1 µg/ml (iii), 0.3 µg/ml (iv), 1 µg/ml (v) or 10 µg/ml mTHPC.

**Supplementary Figure 3.** Representative micrographs showing neurites detected following treatment with mTHPC-mediated PDT in 3D culture then growth for 2 days in the presence of untreated satellite glia. Images show typical neurites in control samples (i), light-only samaples (ii) and mTHPC-PDT treatments of 3 µg/ml (iii), 4 µg/ml (iv) and 10 µg/ml (v).

**Supplementary Figure 4.** A: Fluorecence micrographs showing neurons and satellite glia in mixed neural cultures. All nuclei are labelled blue with Hoechst (i), satellite glia are identified by S100 immunoreactivity with green fluorescence (ii) and neurons are identified by βIII-tubulin immunoreactivity with red fluorescence (iii). The merged image (iv) shows that there were no cells present in these cultures which were not either satellite glia or neurons.

B: Fluorescence micrographs to illustrate the live/dead and immunostaining approach used to quantify the relative sensitivity of cells to PDT. All nuclei are labelled blue with Hoechst, dead nuclei are labelled red with propidium iodide and βIII- tubulin immunopositive neurons are green. Note these are 2D views within the 3D volumes of gel which were assessed manually, these images were not used in the quantification process. (i) shows a dead neuron after treatment with hypericin-PDT, (ii) and (iii) show mixtures of live and dead satellite glia and MCF-7 cells respectively.
